# Extraction Method Effects on Structural Properties and Functional Characteristics of Dietary Fiber Extracted from Ginseng Residue

**DOI:** 10.3390/molecules29204875

**Published:** 2024-10-14

**Authors:** Xiaoyu Feng, Kashif Ameer, Karna Ramachandraiah, Guihun Jiang

**Affiliations:** 1 School of Public Health, Jilin Medical University, Jilin 132013, China; fengxiaoyu0623@163.com; 2 Institute of Food Science and Nutrition, University of Sargodha, Sargodha 40100, Pakistan; kashifameer89@gmail.com; 3 Louisiana State University Health Sciences Center, New Orleans, LA 70112, USA; kramac@lsuhsc.edu

**Keywords:** dietary fiber, ginseng, extraction methods, structure, functional characteristics

## Abstract

In this research, the dietary fibers (DFs) from ginseng residue were extracted by employing three different extraction methods (alkaline: AL, acidic: AC, enzymatic: EN). The extracted DFs were characterized in terms of their structural and functional properties. The results clearly showed that, regardless of the extraction methods, all DF samples exhibited representative infrared spectral features. The DF extracted by AC (citric acid) had more porous structures with a looser configuration, in conjunction with high apparent viscosity, whereas the DF extracted by EN (α-amylase and protease) exhibited higher thermal stability. Moreover, the monosaccharide composition of the DF samples was significantly influenced by the extraction method type. The DF from ginseng residue extracted by AC had the highest functional properties, such as water holding capacity (8.16 g/g), oil holding capacity (3.99 g/g), water swelling capacity (8.13 g/g), cholesterol-absorption capacity (12.85 mg/g), bile acid absorption capacity (91.51 mg/g), nitrite ion absorption capacity (124.38 ug/g at pH 2.0), glucose absorption capacity (52.67 mg/g at 150 mmol/L), as compared to those of DF extracted by the EN and AL (sodium hydroxide) methods. Hence, ginseng residue-derived DF extracted by the AC method may be potentially employed in the preparation of functional food ingredients.

## 1. Introduction

Ginseng (*Panax ginseng* C.A. Meyer), a perennial plant from the Araliaceae family, is typically found in the hilly regions of Eastern Asian countries, such as China, Siberia, and Korea. Ginseng has been used as a medicinal plant in the Traditional Chinese Pharmacopeia because of its medicinal and therapeutic effects. Ginseng root has been recognized for millennia to provide health benefits, and it is commonly used in the creation of supplements and herbal treatments. Ginseng has a variety of health advantages, including improved cognitive performance, increased general well-being, and relief from physical and emotional stressors [1,2]. Ginsenosides, polysaccharides, volatile oil, and other active components are currently extracted exclusively by ginseng processing. However, ginseng residues high in dietary fiber (DF) are wasted, which not only depletes resources but also pollutes the environment. Ginseng residues are the plant elements that remain after extracting the bioactive compounds from the ginseng root. This includes fibrous fibers, residual ginsenosides, and other phytochemicals that may be beneficial to health. These residues, which are often seen as waste, may really be abundant in nutrients and bioactive compounds.

DFs are the total of non-starch polysaccharides that are resistant to enzymatic digestion in the small intestine [3]. DFs are composed of soluble dietary fiber (SDF) and insoluble dietary fiber (IDF) [4]. In general, SDF is obtained by treating the supernatant derived from enzymatic, chemical, or mechanical processing methods with ethanol, while IDF is obtained by washing the precipitate. SDF demonstrated not only a beneficial physiological function, but also a greater ability to produce viscosity and form gels, whereas IDF raises stool volume, encourages defecation and prevents obesity [5,6,7,8]. Ginseng DFs have various health benefits, including a lower risk of diseases such as diabetes, colorectal cancer, and cardiovascular disease [9], in addition to their use as nutritional supplements. Ginseng DFs are also employed as vaccine adjuvants [10].

Currently, diverse extraction techniques, including chemical, enzymatic, and fermentation procedures, are utilized to extract dietary fibers from various sources. Various processing conditions of distinct extraction methods may modify the structural and functional features of dietary fibers, hence affecting their functional and physicochemical qualities [11,12,13,14].

Commonly used methods for the extraction of fibers include gravimetric, microbial, enzymatic and chemical methods. Advances in technology have led to the development of techniques such as high hydrostatic pressure and ultrasound-based methods. However, the chemical method and the enzymatic method stand out for their simplicity in operation and minimal energy consumption. An alkaline (AL) solution proves effective in breaking down the glycosidic bond present in DFs, and acid (AC) extraction can effectively hydrolyze hemicellulose, thereby changing the ratio of SDF and IDF [15,16]. By contrast, enzyme (EN) extraction can destroy cellulose, hemicellulose, and lignin in the cell wall, contributing to the conversion of IDF to SDF [17]. However, there are currently few reports on the utilization of AL, acidic AC, and EN extraction methods for ginseng residue DFs. Hence, the AL, AC, and EN extraction methods were used for DF extraction from the ginseng residue. Furthermore, ginseng residue DF fractions obtained from various methods were compared with respect to structural attributes. Moreover, the proximate composition and functional properties were also assessed. This research could provide insights related to ginseng residue DF and offer a theoretical basis for ginseng residue extraction and processing for developing functional food ingredients.

## 2. Results and Discussion

### 2.1. Extraction Yield and Proximate Composition

Ginseng residue DFs extracted by enzymatic (G-EN), acidic (G-AC), and alkaline (G-AL) methods were analyzed for total DF yield and proximate composition. The results are shown in Table 1. Among all DF extracts, the highest DF yield was exhibited by the G-AL (74.78%), followed by G-EN (67.96%) and G-AC (60.53%). The highest DF yield from the alkaline method might be ascribed to the disintegration and destruction of the cellular matrix, which caused the disruption of molecular compactness in the cells’ structural configuration after exposure to highly alkaline conditions. This alkaline extraction caused hemicellulose partial dissolution in IDF, which resulted in the obtainment of a higher DF extraction yield [18]. In terms of composition, G-AC had the highest protein (2.10%), ash (0.31%), and fat (0.22%) contents among all samples. Feng et al. [19] and Wang et al. [20] have also reported similar findings pertaining to the DF yield and proximate composition from papaya (peel and seed) and kiwi fruit by alkaline, enzymatic, and acidic extraction methods.

### 2.2. Microstructural Properties

**Figure 1:**
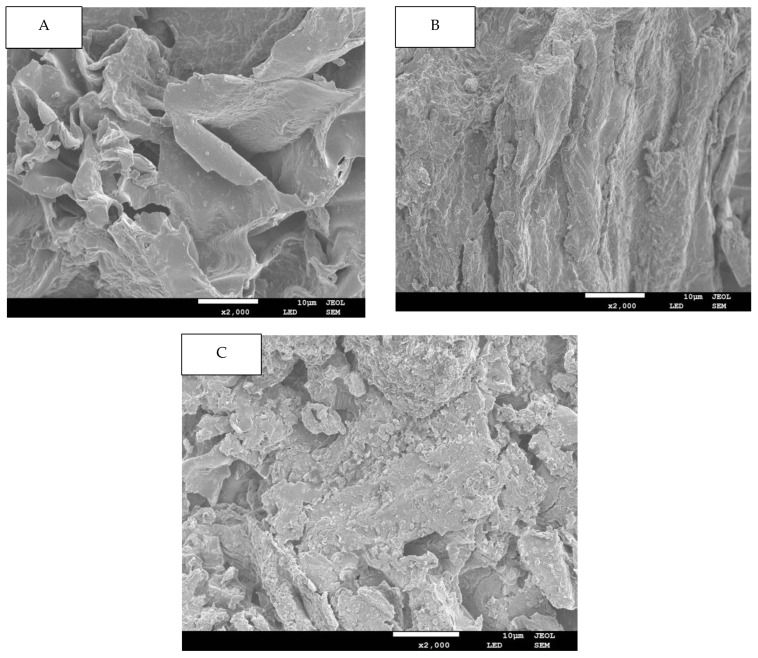
SEM images for G-AC (**A**), G-AL (**B**), and G-EN (**C**).

In this study, the microstructural attributes of all DFs fractions were elucidated by SEM and are demonstrated in Figure 1 for of G-AC (A), G-AL (B) and G-EN (C). In the case of the G-AC samples, the DF’s surface exhibited prominent surface irregularity, enhanced pore loosening, cell-wall disintegration, as well as microstructural impairment as compared to the surface of the DF extracted by G-EN and G-AL. The DFs extracted by the enzymatic method had more complicated structures, in conjunction with loosened pores and surface roughness, while the G-AL showed a relatively flat structure. This could possibly be ascribed to the fact that sodium hydroxide (NaOH) caused potent oxidation during the extraction process. The high degree of structural impairment in the case of acidic fractions could be due to the loosening and disintegration of the DF’s sheet-like structure. It was also evident from the SEM micrographs that the acidic method significantly affected the ginseng residue-derived DF structure. Furthermore, DFs with looser spatial structures and less degree of compactness exhibited an enhanced surface area and may exert a significant effect on their adsorption capacities, such as oil and water retention, bile acid, nitrite ion, and glucose absorption capacities. Conclusively, it could be inferred that the extraction method type may cause significant alterations in the properties of DFs extracted from ginseng residue.

### 2.3. FTIR Spectroscopy

**Figure 2:**
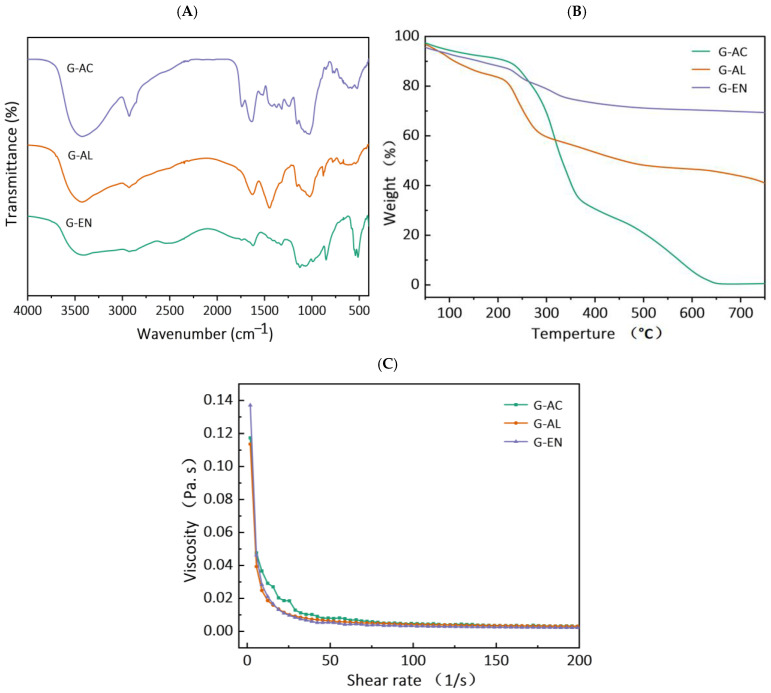
FT-IR spectra (**A**), thermal properties (**B**), and rheogram plot (**C**) for G-AC, G-AL, and G-EN.

The transmittance percentages of all extracted DF samples from ginseng residue were calculated. The results are shown in Figure 2A. Two broad peaks are visible in all DF fractions at the IR spectral regions of 3427 and 2927 cm^−1^, and these stretching vibrations possibly indicate the presence of –CH of sugar methyl, hydroxyl (-OH), and methylene groups, respectively [21]. Wide absorption peaks are also visible at the IR regional range of 1200–1600 cm^−1^, which might be attributable to the variable angular vibrational stretching of CH bonds. Moreover, the appearance of absorption peaks at these characteristic spectral IR regions has been reported in published reports corresponding to saccharides in DF fractions [20]. In a similar manner, regardless of the extraction method employed for extracting DFs, all DF samples showed the presence of IR spectral peaks at IR regions ranging from 1000 to 1350 cm^−1^, and this may be ascribed to the contraction of IR vibrations, possibly indicative of the presence of C–O ester bonds [22]. Furthermore, the IR spectral regions ranging from 700 to 1100 cm^−1^ are most likely indicative of α- and β-pyran monosaccharides. Relatively weak intensity IR peaks were found at IR regions ranging from 3414 to 3480 cm^−1^ and from 2856 to 2923 cm^−1^, which evidently suggests possible stretching vibrations of –OH and C–H groups pertaining to polysaccharides polymers in all DF’s fractions. This might be explicable because of the possible disintegration of molecular configuration and the breaking of intermolecular bonds after extraction by enzymatic, acidic, and alkaline methods. The FTIR results showed that enzymatic extraction in the DF samples caused less IR peak broadening in comparison with those of acidic- and alkaline-extracted DF fractions. They also imply that the enzymatic treatment caused severe hydrolysis, followed by the alkaline extraction method, in the case of pectin and hemicellulose of the DF derived from ginseng residue. Among all DF samples, the characteristic IR absorption peak at IR spectral region of 1000 cm^−1^ was indicative of the possible presence of the C–O group in C–O–C bonding (a typical xylan IR peak), which implies that the DF samples’ structural configuration comprised xylan hemicellulose. Furthermore, the stretching/bending vibration of aromatic lignin hydrocarbons corresponded to the spectral region at 1660 cm^−1^. However, this characteristic IR peak was not visible in the DF samples extracted by enzymatic and alkaline treatments, implying that the alkaline and enzymatic treatments caused increased disintegration in the molecular configuration of lignin. The results of this study are in agreement with the previous published report by Wang et al. [20] on DF derived from kiwi fruit.

### 2.4. Thermal Properties

TGA analysis was carried out and Figure 2B shows the TGA curves for all DF samples. The TGA thermogram curve involves three stages. The initial stage usually occurs within a temperature range of 30–210 °C, and at this stage, regardless of the extraction method, the DF samples exhibited a decrease in weight loss, accompanied by the occurrence of devolatilization at 120 °C. Among all samples of ginseng residue-derived DFs, the greatest decrease in percent weight loss was found in the G-AC samples, followed by the G-AL and G-EN samples, with the corresponding gradual rise in temperature. During the first TGA stage, the declining trend of percent weight loss may be ascribed to the possible evaporation of absorbed water from each extracted DF sample. The results also indicated that the second stage of the TGA curve was found within the temperature range of 210–400 °C. The maximum levels of percent weight loss occurred during this second stage of TGA analysis. During the second stage, this highest level of percent weight loss might be explicable with respect to the high degree of degradation of pyrolytic polysaccharides, which primarily comprised hemicellulose and pectic polysaccharides. The percent weight loss exhibited a significantly (*p* < 0.05) lower trend in G-EN, while G-AC and G-AL exhibited higher percent weight loss. This implies that the DFs extracted by the EN method were relatively more thermally stable as compared to the G-AC and G-AL fractions. The G-EN samples exhibited a lower percent weight loss, which implies that exposure to the EN treatment led to less cleavage of the polymer chain, compared to the other DF fractions. The probable reason for the increased thermal stability of the DFs extracted by the enzymatic method is enhanced structural modification. Moreover, the extracted DFs polysaccharides were not degraded in G-EN when compared with G-AC and G-AL. Furthermore, the declining tendency of percent weigh loss in G-EN, as compared to the other samples, might be attributable to several factors pertaining to the nature of the DF obtained and the type of extraction method utilized. The probable reasons for this phenomenon might be the following: (1) the extraction conditions for enzymatic conditions are relatively milder compared to alkaline and acidic conditions, and the use of enzymes is relatively more selective and specific, which allows for the targeted degradation of cell wall components without exerting any detrimental effect on the extracted DF; (2) the structural integrity of DF is less likely to be disrupted after exposure to enzymatic treatment; AL and AC conditions may also cause chemical modification owing to DF structural breakdown, which enhances susceptibility to thermal degradation; (3) AL and AC treatments may also lead to the presence of chemical residues during the extraction process, which may cause thermal weight loss during TGA analysis [23,24]. EN extraction involves less deposition of chemical residues that may play a contributory role in thermal degradation. The results of this study are in agreement with previously published reports by Feng et al. [19], who reported thermal weight loss in papaya peel and seed DF.

### 2.5. Viscosity

All DF samples were subjected to measurement of viscosity as a shear rate function, and the results are presented in Figure 2C. All DF samples were analyzed for the consistency coefficient, apparent viscosity, and flow behavior index, and the results are tabulated in Table 2. Irrespective of the extraction method used for DF extraction, all samples exhibited similarity with respect to the rheograms. The decreasing viscosity tendencies as a function of shear rate in the case of the G-EN and G-AL samples were more prominent compared to those of G-AC. The apparent viscosities for G-AC, G-EN, and G-AL were 18.54, 9.68, and 10.06 m.Pa.s, respectively (Table 2). The apparent viscosities of G-EN and G-AL were comparable to each other, whereas the highest apparent viscosity among all samples was exhibited by G-AC. The AC extraction increased the effective area of the fiber surface (Figure 1) and resulted in more particle interactions and an enhanced network structure with water molecules, resulting in a significant increase in apparent viscosity [25]. The consistency coefficients for the G-AC, G-EN, and G-AL samples were recorded as 182.39, 244.09, and 178.62 m.Pa.s, respectively. The flow behavior index values for G-AC, G-EN, and G-AL were 0.26, 0.05, and 0.19, respectively. It is noteworthy that hydrogen bonding linkages and charge-transfer complexes in the DF polymer chains significantly affected (*p* < 0.05) the viscosity of the DF samples. G-AC with high viscosity could be used as a thickener, gelatinizing agent, texture modifier, suspending agent, and stabilizer for applications in food manufacturing [26].

### 2.6. Monosaccharide Composition

**Figure 3:**
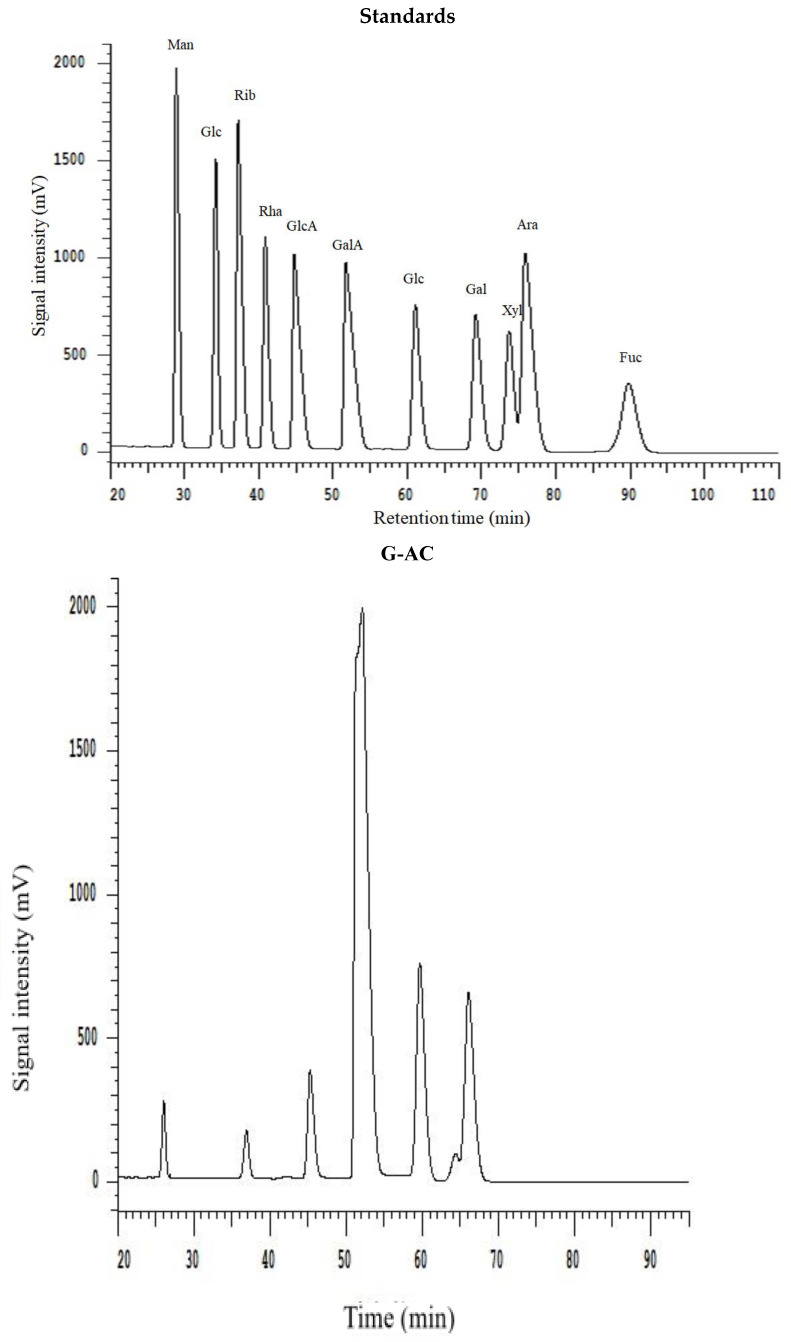

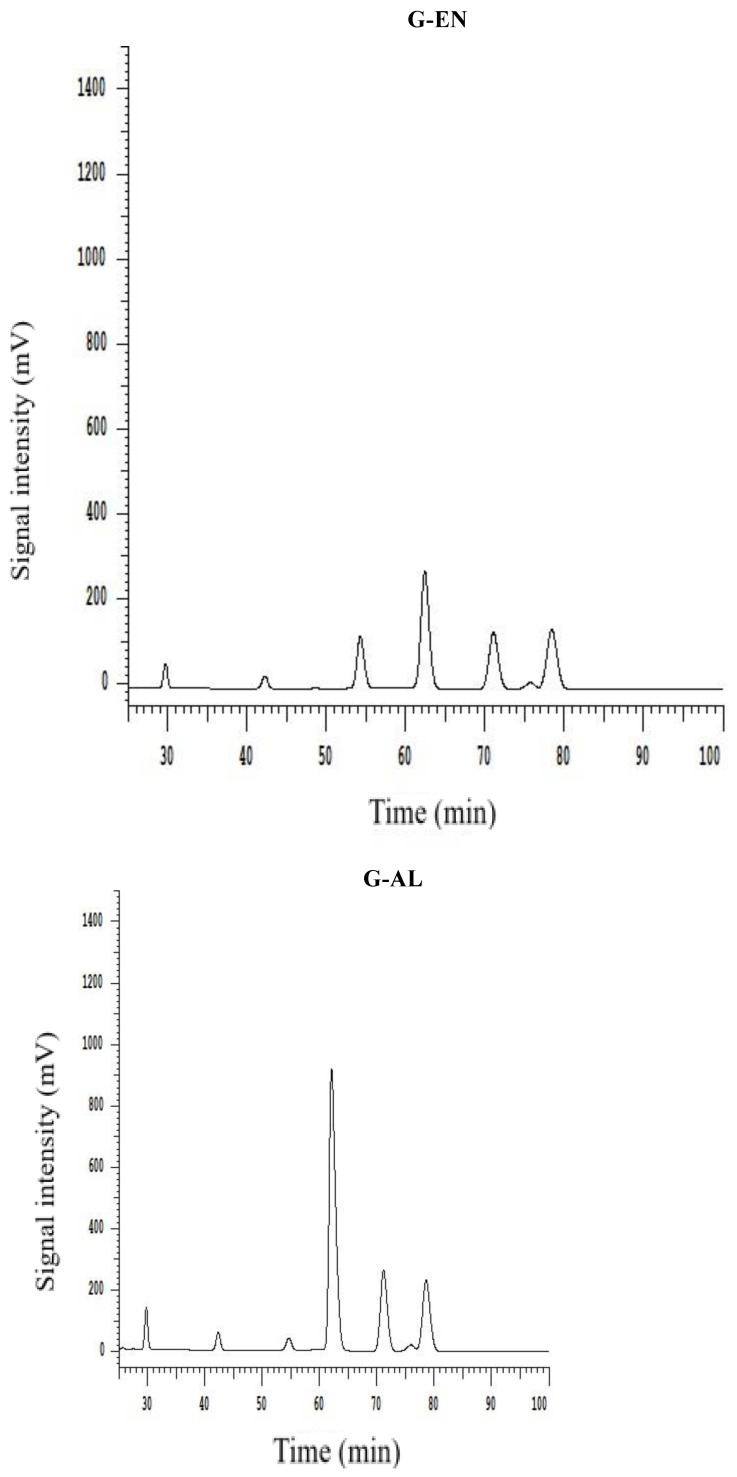
HPLC chromatogram of monosaccharide standards and monosaccharide compositions for G-AC, G-AL, and G-EN.

All extracted DF samples were analyzed for monosaccharide composition by HPLC, and the results are provided in Table 3. The HPLC chromatograms are depicted in Figure 3. The results show that the DFs samples exhibited the presence of different monosaccharides, such as galacturonic acid, mannose, rhamnose, galactose, glucose, arabinose, and xylose. The most abundantly found monosaccharides in the ginseng residue DF samples were galacturonic acid, galactose, and glucose. It has already been reported in previously published reports that DF comprises the main monosaccharides such as galactose, arabinose, and glucose, from DFs of sweet potato and calamondin pomace [27,28]. Starch and cellulose mainly serve as the precursor molecules for the formation of glucose in DFs. G-AC, G-EN, and G-AL exhibited glucose molar ratios of 74.02, 26.70, and 59.69, respectively. G-AC had the highest molar ratio of glucose, followed by G-AL, which may be possibly attributable to the enhanced degree of hydrolysis of cellulose in the DF cell wall after exposure to acidic treatment. Furthermore, G-AC exhibited higher molar ratios of galactose (24.35) and arabinose (18.49) compared to G-AL and G-EN. High molar ratios of galactose and arabinose could be implied as the main contributory factor to the increased SDF content [29], which in turn influenced the WHC of ginseng residue DF samples. The galacturonic acid molar ratio was the highest in G-AC (9.86), followed by G-EN (7.78) and G-AL (1.89), respectively. Galacturonic acid has been reported in published literature as one of the components of pectin found in plant-based DFs [20]. The highest galacturonic acid in the case of G-AC and G-EN was indicative of a high content of pectin in the SDF fraction. Pectin comprises neutral sugars with varying amounts, such as L-arabinose, L-rhamnose, D-galactose, and D-xylose. Moreover, a few of the neutral sugars serve as the main building block or constituents of galacturonan backbone side chains [30]. In the case of rhamnose and mannose, similar molar ratios were found by the G-AC G-AL and G-EN methods. On the basis of the findings of this study, it might be implied that a significant influence (*p* < 0.05) on monosaccharide composition was exhibited by different extraction methods, especially the galacturonic acid, glucose, galactose, and arabinose molar ratios in the DF samples.

### 2.7. Hydration Properties

The hydration properties, such as WHC, OHC, and WSC, were measured for all extracted DF samples, and the results are tabulated in Table 4. The water retention capacity of foodstuffs is usually denoted as WHC and is measured after being subjected to various processing operations, centrifugation, and compression. In terms of WHC, G-AC had the highest WHC of 8.16 g/g, followed by G-EN (4.62 g/g) and G-AL (2.40 g/g). WHC refers to the proportionate amounts of water trapped in physical manner, linked water, and hydrodynamic water. Moreover, as an inherent characteristic of foodstuffs, WHC has been correlated with diverse DF surface areas, densities, and structures, as well as with hydrophilic sites. In general, DF samples with higher WHC values may exhibit viscosity alteration in conjunction with the prevention of food shrinkage. This results of this study were found to be in accord with findings reported by Wang et al. [20] and Gan et al. [17] regarding DF extracted from kiwi fruit and grapefruit peels, respectively.

OHC has been defined in the published literature as the natural DF’s capacity for maintaining oil droplets after the application of shear mixing and centrifugation to the extracted DF. OHC, as a functional property, is significant in food manufacturing for preventing the loss of fat during cooking. Regarding the OHC, the G-AC samples had the highest OHC of 3.99 g/g, followed by G-EN (1.58 g/g) and G-AL (1.37 g/g). The high OHC value of G-AC could be attributed to the DF structural loosening phenomenon after exposure to acidic treatment. Moreover, it could be inferred that foods fortified with ginseng residue DFs could be utilized as a unique source for the preparation of related food products. These may also play a significant role in the prevention of water syneresis during the formulation of foodstuffs and may also act as emulsifiers because of their potentially high OHC and WHC, making them suitable for manufacturing high-fat foods [31,32].

In the published literature, WSC is described as the ratio of DF volume when immersed in an excess of water after achieving equilibrium and the actual weight of DF. Generally, two mechanisms of water and DF interaction have been reported: (1) retention of water molecules, possibly owing to the formation of dipoles and hydrogen linkages, and (2) retention of water by capillary structures as a result of surface tension strength. Moreover, particle sizes and the presence of free polar groups may also exert significant influences on the enhancement of DFs’ WSC. In the case of WSC, the G-AC samples had the highest WSC of 8.13 g/g, followed by G-EN (5.30 g/g) and G-AL (3.23 g/g) (Table 4). The most probable explanation for the highest WSC in the case of G-AC might be explained by the fact that WSC has been reported to have a positive correlation with the pectin content. It is evident from the results that acidic treatment by means of citric acid led to less degradation of pectin in the ginseng residue DFs in comparison with alkaline extraction through NaOH solution. The AL treatment caused severe degradation and disintegration of pectin in the DF cell wall, leading to the declining tendency in the WSC of G-AL samples, as compared to G-AC and G-EN. These results were found to be in line with the results reported by Ma and Mu [33], in which the authors concluded that different extraction methods exhibited significant effects on the WSC of DF extracted from deoiled cumin.

### 2.8. Adsorption Capacities

#### 2.8.1. Nitrite Ion Absorption Capacity (NIAC)

The DF samples were analyzed for NIAC values, which were recorded at two different pH levels of 2.0 and 7.0. The results are given in Table 5. Nitrite (NO^2−^) ions may lead to the onset of risk factors of cancer, accompanied by fetal malformations under gastric acid conditions [17]. Among all DF samples, the G-AC samples had the highest values of NIAC at both pH levels (2 and 7). At pH 2.0 and 7.0, the NIAC values of all DF samples ranged between 121.45 and 124.38 ug/g and between 117.32 and 120.47 ug/g, respectively. The NIAC values of both G-AC and G-EN samples were comparable to each other at both pH levels of 2.0 and 7.0, whereas G-AL had the lowest values of NIAC at both pH levels. In this research, the G-AC samples had the highest NIAC of 124.38 ug/g, which was significantly lower (*p* < 0.05) than the NIAC values of kiwifruit SDF (138 ug/g) and SDF from the grapefruit peel (219.43 μg/g) [17,20]. Moreover, it may be implied from the results that the NIAC values of the DFs extracted from ginseng residue were dependent on the type of extraction method employed, suggesting that the DF derived from ginseng residue involved the potential absorption of the nitrite ions, which are then excreted through feces.

#### 2.8.2. Bile Acid Absorption Capacity (BAC)

All DF samples were analyzed to determine the BAC values, and the results are presented in Table 5. Usually, the liver is the place where the biosynthesis of bile acids occurs by using cholesterol as the precursor molecule. It has been reported in previously published reports that DFs have the capacity to bind bile acid, which leads to the enhanced elimination of bile acid and contributes to an increased tendency to convert to cholesterol-bound acid, hence leading to decreases in serum cholesterol levels and a reduced incidence of cardiovascular diseases [34]. Among all DF samples, G-AC had the highest BAC value (91.51 mg/g), followed by G-EN (89.39 mg/g) and G-AL (84.31 mg/g). The results showed that the extraction method type exerted a significant (*p* < 0.05) effect on the BAC values of the extracted DF samples. It has also been reported in previously published reports that the gel properties and the anionic group content of DFs could influence the BAC of DFs [34,35]. Moreover, the DFs’ capacity to bind bile acids has been linked to the DFs’ surface characteristics and internal structural configuration [11].

#### 2.8.3. Cholesterol Absorption Capacity (CAC)

The CAC values for all DF samples were evaluated, and the results are presented in Table 5. CAC measurement is defined in terms of the total cholesterol estimated to be absorbed by DF. Regarding the physical absorption of cholesterol, several factors, including porosity, particle size, surface area, and temperature, play significant contributory roles. With respect to chemical absorption, electrostatic charges may exert significant effects. The DF’s tendency to absorb cholesterol may lead to a decreasing trend in serum cholesterol levels. Among all DF samples, the highest CAC value was found in G-AC (12.85 mg/g), whereas both G-EN and G-AL had comparable CAC values of 12.13 mg/g and 11.13 mg/g, respectively. The G-AC samples had increased CAC values, which might be attributable to the loosening of the DF’s structural framework after exposure to AC treatment by means of citric acid. The results suggested that the AC treatment to extract ginseng residue DF led to an increased interaction between the hydrophilic groups and the water molecules, which consequently caused increases in WHC and WSC. The increased CAC of G-AC might be attributable to the high degree of porosity in the DFs’ structural configuration. Additionally, the higher CAC values for the G-AC samples may also be because of the presence of a honeycomb structure on the surface of the smaller particles, which probably enhanced the binding of cholesterol by DFs.

#### 2.8.4. Glucose Absorption Capacity (GAC)

The GAC values of different DF samples were assessed, and the results are given in Table 5. The estimation of GAC values was carried out at a glucose concentration range of 50–150 mmol/L. Glucose is usually found in the gastrointestinal juice, and DFs exhibit glucose-binding ability and may cause a decreasing trend in postprandial serum glucose levels. Hence, GAC is described as one of the significant functional characteristics of DFs. Furthermore, previously published studies have reported on the phenomenon of the increasing trend of GAC, which might be ascribed to the enhanced surface area and the presence of a large number of cavities. Comparatively, at a glucose concentration range of 50–150 mmol/L, G-AC exhibited the highest GAC values, ranging from 18.68 to 52.67 mg/g, among all samples, as compared to those of G-EN (17.64–50.68 mg/g) and G-AL (17.21–48.67 mg/g) samples. Comparatively at glucose concentration levels of 50 mmol/L and 100 mmol/L, both the G-EN and G-AL samples had comparable values of GAC. However, the G-EN samples had slightly higher GAC values at a glucose concentration of 150 mmol/L, as compared to the G-AL samples. Conclusively, it may also be inferred based on the results that the extraction method type exerted a significant (*p* < 0.05) effect on the GAC values of ginseng residue DFs, and the extracted DF samples had in vitro hypoglycemic potential.

## 3. Materials and Methods

### 3.1. Materials

The procurement of dried ginseng was carried out at an indigenous supermarket located in Jillin city, China. The ginseng cultivated (Latitude 43°50′46″ N, Longitude 126°33′42″ E) for a period of 5 years was considered for sample collection. All the reagents and chemicals employed to carry out this study were of analytical grade.

### 3.2. Ginseng Residue Preparation

The method reported by Jiang et al. [36] was used to prepare ginseng residue. To extract the ginseng polysaccharides, a boiling procedure was carried out at a temperature of 102 °C + 5 °C, which led to ginseng residue formation. Then, the washing of ginseng residue was performed with ethanol, followed by distilled water, to eliminate inorganic salts and oligosaccharides (water-soluble). Then, the dried residue was packaged and stored until further analysis.

### 3.3. Enzymatic (EN) Extraction Method

Ginseng residue DF was extracted by the EN extraction method reported by Kurek et al. [37]. Ginseng residue was taken in a specified amount (10 g), followed by gentle mixing with phosphate buffer (160 mL, pH 6.8, 20 mM). Afterwards, 0.1 g of heat-stable α-amylase was added into the reaction mixture and allowed to prepare a stir-aided suspension. A shaking water bath was employed for the incubation of the ginseng residue sample at 75 °C until a negative iodine test was observed, which took a time period of 30 min. Afterwards, the suspension was subjected to cooling at 60 °C, and then suspension pH was adjusted up to 7.5. Then, a protein digestion procedure was carried out by mixing with the protease enzyme (200 μL), and then the reaction mixture was allowed to stand for a 30 min time interval in the shaking water bath. Then, centrifugation was performed for a 15 min time interval at 5000× *g*, followed by residue extraction using distilled water with double rinsing. The oven-drying of samples was then carried out at 50 °C to complete the extraction of ginseng IDF. Meanwhile, the collected supernatants were then mixed with 95% ethanol (four-fold volume and 2 h time interval) at a room temperature of 25 °C. Then, SDF was obtained from the ginseng residue by drying under the fume hood. Then, the mixing of both IDF and SDF extracts was carried out prior to further analysis. Ginseng residue DFs extracted by EN are referred to as G-EN.

### 3.4. Acid (AC) Extraction

An amount of 400 mL of 1% *w*/*v* citric acid was accurately measured and utilized for AC treatment to obtain the AC fraction of DF from ginseng residue [38]. For AC extraction, 400 mL of AC solution was added to 10.0 g of ginseng residue, and the resultant solution mixture was subjected to a 2 h extraction in a water bath at 40 °C. Then, the AC fraction of ginseng residue was obtained as per the methodology described in Section 2.3. Ginseng residue DFs extracted by AC are referred to as G-AC.

### 3.5. Alkali (AL) Extraction

Ginseng residue DF was subjected to alkali extraction by using a 5% NaOH (*w*/*v*) solution [20]. For AL extraction, the NaOH solution (400 mL) was mixed with ginseng residue in a specified amount (10.0 g), followed by a 2 h time interval in the water bath at 40 °C temperature. Then, the AL fraction of ginseng residue was obtained according to the methodology mentioned in Section 2.3. Ginseng residue DFs extracted by AL are referred to as G-AL.

### 3.6. Proximate Composition

All DF fractions were analyzed to determine their proximate composition, including protein, fat, and ash contents, according to AOAC methods [39]: fat (AOAC Method 996.06), protein (AOAC Method 930.15), and ash (AOAC Method 942.05).

### 3.7. Scanning Electron Microscopy (SEM)

The effects of extraction methods were observed in the microstructural and morphological attributes of DF, as examined by scanning electron microscopy (SEM: SU8010, Hitachi, Tokyo, Japan), employed for microstructural elucidation. The DF from each ginseng residue sample was specifically attached to a specimen holder. Then, each sample was plated with gold powder using a sputter coater. The samples were then subjected to SEM analysis at operation parameters of 5 kV voltage, during which the images were captured at the magnification level of 2000×.

### 3.8. Fourier Transform Infrared (FTIR) Spectroscopy

The organic functional groups of the DF samples were analyzed by FTIR spectroscopy. For FTIR analysis, an FTIR spectrophotometer (Tensor 27, Bruker Daltonics Inc., Bremen, Germany) was employed. The dried DF samples were ground with KBr (1:100, *w*/*w*), followed by pressing to pellets. The FTIR analysis was carried out at the spectrum wavelength range of 400–4000 cm^−1^. The speed of the scan used for analytical purposes was <10 s, at resolution of 4 cm^−1^ for 32 scans. Finally, the FTIR spectra were obtained by carrying out spectroscopy in Attenuated Total Reflection (ATR) mode.

### 3.9. Thermal Properties

In this study, the thermal properties of the DFs samples were assessed using a thermogravimetric analyzer (TG/DTA 8122, Rigaku, Tokyo, Japan). Under an inert nitrogen environment, the thermogravimetric measurement was carried out at an operational temperature ranging from 30 to 600 °C, accompanied by a heating rate of 20 °C/min.

### 3.10. Viscosity Measurements

The DF solutions (0.1% dry weight/volume) were prepared by dissolving each ginseng residue fiber in a 20 mM sodium phosphate buffer (pH 6.5). Residues were stirred at ambient temperature for 10 min, followed by stirring at 80 °C for 15 min. The viscosities of the prepared DF solutions were measured using a rheometer (Waters Discovery HR-1, TA, New Castle, DE, USA) with steel cone geometry C60/1°. The measurements were performed at 25 °C over a shear rate range of 0 to 200 s^−1^. The apparent viscosity (γ^−^), flow behavior index (n), and consistency coefficient (K) of the DF samples were measured as described by the Power Law model (η = Kγ ^n−1^), following the previous method detailed by Jiang et al. [10]. In the above equation, η indicates the apparent viscosity (Pa·s^−1^), K denotes the consistency index (Pa·sn), while n indicates the flow behavior index (dimensionless).

### 3.11. HPLC Determination of Monosaccharides

The DF from each extraction method was analyzed for monosaccharide composition by high performance liquid chromatography (HPLC) technique according to Jiang et al.’s [10] method. A DF sample from each extraction method was taken in a specified amount of 2 mg and subjected to dissolution with trifluoroacetic acid (TFA) (1 mL: 2 M). This reaction mixture was prepared at 120 °C under sealed conditions in a hydrothermal reactor for a 2 h time interval. Before performing the HPLC analysis, the aqueous layer was removed by employing a 0.45 µm standard filter. Dionex Thermo Ultimate 3000 HPLC (Dionex Co., Sunnyvale, CA, USA) was used for monosaccharides detection. During this HPLC analysis, a diode array detector (DAD), sourced from Thermo Fisher Scientific, was used for monosaccharide detection. Preparation of the mobile phase involved the mixing of the phosphate buffer solution (PBS, pH 6.7, 0.1 mol/mL) and acetonitrile (A) in a ratio of 82:18 (*v*/*v*). HPLC analysis was carried out using a Supersil ODS2 column (4.6 × 250 mm^2^; 5 µm). The injection volume and flow rate were 20 µL and 0.8 mL/min, respectively. Against reference standards, the analysis was completed at detection wavelength of 245 nm.

### 3.12. Water Holding Capacity (WHC) and Oil Holding Capacity (OHC)

The reported method described in a published report by Raza et al. [40] was used for the measurement of WHC and OHC. In brief, each extracted DF sample was weighed (0.5 g), and then samples were transferred to a centrifuge tube. Distilled water in an amount of 5 mL was added to each centrifuge tube, and then incubation of each samples was performed at 37 °C incubation temperature for time interval of 1 h. After completion of the incubation period, the water was removed by centrifugation, and measurement of the WHC of samples was carried out according to Equation (1), given below.

(1) $$WHC\;\left(\frac{g}{g}\right)=\frac{W_{2}-W_{1}}{W_{1}}$$

where the total sample weight is denoted by *W*_1_ on the basis of dry weight. Conversely, the weight of the total samples was calculated after filtering out the water from the samples and is represented by *W*_2_.

All extracted samples of DF were analyzed for their OHC. The mixture of each DF sample from various extraction methods was prepared by combining the samples. The preparation of the sample mixture involved transferring an accurately weighed amount (0.5 g) of the sample in a centrifuge tube, to which soybean oil was then added. The reaction mixture in the centrifuge tube was allowed to incubate (37 °C temperature and time interval of 1 h). The centrifugation of samples was carried out at 5000× *g* for a time period of 10 min. The OHC was calculated by using Equation (2), given below:

(2) $$OHC\;\left(\frac{g}{g}\right)=\frac{W_{2}-W_{1}}{W_{1}}$$

where *W*_1_ in the above equation represents the total sample weight based on dry weight. After removing the excessive soybean oil, the total sample weight was calculated and is represented by *W*_2_.

### 3.13. Water Swelling Capacity (WSC)

All DF samples were analyzed for the WSC as per the He et al. [41] reported method. Each DF sample was accurately weighed (0.5 g) and then transferred to the measuring cylinder (25 mL). This reaction mixture was then mixed with the distilled water in an amount of 20 mL. The volume measurement was carried out prior to and after completion of time interval allowed for standing. Then, WSC measurement of the DF dry sample was performed in terms of volumetric change according to Equation (3), given below:

(3) $$WSC\;\left(\frac{mL}{g}\right)=\frac{V_{2}-V_{1}}{m_{0}}$$

where the dry weight of each sample is represented by *m*_0_, whereas the initial volume is depicted by *V*_1_. *V*_2_ represents the final volume, which was measured after the completion of the standing time interval.

### 3.14. Adsorption Capacities

The nitrite-ion adsorption capacity (NIAC), the bile acid adsorption capacity (BAC), the cholesterol adsorption capacity (CAC), and the glucose adsorption capacity (GAC) were determined according to the methods described by Luo et al. [42] and Wang et al. [20], respectively.

### 3.15. Statistical Analysis

SPSS 20.0 software (SPSS Inc., Chicago, IL, USA) was used to perform all the statistical analyses. The triplicate manner was adopted for all performed experiments. The results are presented as the mean with standard deviation (SD). For evaluating the differences between the means, an analysis of variance (ANOVA), accompanied by Duncan’s multiple range test (DMRT), were employed at a significance level of *p* < 0.05.

## 4. Conclusions

This study aimed to investigate the structural and functional characteristics of DFs extracted from ginseng residue using the alkaline, acidic, and enzymatic methods. Among all DF extracts, the highest DF yield was exhibited by the G-AL (74.78%), followed by G-EN (67.96%) and G-AC (60.53%). G-AC had more complicated structures, in conjunction with loosened pores and surface roughness. The extraction method type not only significantly influenced the structural characteristics of DF fractions from each extraction method but also exerted significant effect on the DFs’ functional properties. Among the extraction methods, DF extracted by the acidic method demonstrated the highest functional properties, including water holding capacity (WHC), oil holding capacity (OHC), water solubility capacity (WSC), cholesterol absorption capacity (CAC), bile acid adsorption capacity (BAC), nitrite ion adsorption capacity (NIAC), and glucose adsorption capacity (GAC). Therefore, DF extraction is a form of deep processing of ginseng residue, which can lead to a rising trend in the value addition of the ginseng industry.

## Figures and Tables

**Table 1:** DF yield and composition of G-AC, G-AL, and G-EN.

	G-AC	G-AL	G-EN
DF yield (%)	60.53 ± 1.01 c	74.77 ± 0.56 a	67.96 ± 0.39 b
Protein (%)	2.10 ± 0.09 a	0.86 ± 0.01 c	1.41 ± 0.14 b
Ash (%)	0.31 ± 0.01 a	0.16 ± 0.03 b	0.23 ± 0.04 b
Fat (%)	0.22 ± 0.03 a	0.17 ± 0.01 b	0.18 ± 0.01 b

Different letters in the same row for each parameter represent statistically significant differences (*p* < 0.05).

**Table 2:** Apparent viscosity, consistency coefficient, and flow behavior index of G-AC, G-AL, and G-EN.

	G-AC	G-AL	G-EN
Apparent viscosity 25 1/s [γ̇, mPa s]	18.54 ± 0.12 a	10.06 ± 0.17 b	9.68 ± 0.26 b
Consistency coefficient [K, mPa s]	182.39 ± 1.81 b	178.62 ± 2.12 c	244.09 ± 1.54 a
Flow behavior index [n, -]	0.26 ± 0.004 a	0.19 ± 0.001 b	0.05 ± 0.001 c

Different letters in the same row for each parameter represent statistically significant differences (*p* < 0.05).

**Table 3:** Molar ratio of monosaccharide components of G-AC, G-AL, and G-EN.

	G-AC	G-AL	G-EN
Mannose	4.00	3.31	2.48
Rhamnose	3.33	2.72	2.13
Galacturonic acid	9.86	1.89	7.78
Glucose	74.02	59.69	26.70
Galactose	24.35	12.81	10.30
Xylose	1.00	1.00	1.00
Arabinose	18.49	10.72	10.13

**Table 4:** Hydration properties of G-AC, G-AL, and G-EN.

	G-AC	G-AL	G-EN
WHC (g/g)	8.16 ± 0.18 a	2.40 ± 0.13 c	4.62 ± 0.14 b
OHC (g/g)	3.99 ± 0.21 a	1.37 ± 0.06 b	1.58 ± 0.09 b
WSC (g/g)	8.13 ± 0.06 a	3.23 ± 0.15 c	5.30 ± 0.10 b

Different letters in the same row for each parameter represent statistically significant differences (*p* < 0.05).

**Table 5:** Adsorption capacities of G-AC, G-AL, and G-EN.

		G-AC	G-AL	G-EN
NIAC (ug/g)	pH = 2	124.38 ± 0.31 aA	121.44 ± 0.36 cA	123.07 ± 0.13 bA
	pH = 7	120.47 ± 0.49 aB	117.32 ± 0.30 cB	119.43 ± 0.39 bB
BAC (mg/g)		91.51 ± 0.14 a	84.30 ± 0.29 c	89.39 ± 0.19 b
CAC (mg/g)		12.85 ± 0.12 a	11.13 ± 0.19 c	12.13 ± 0.06 b
GAC (mg/g)	50 mmol/L	18.68 ± 0.12 aC	17.21 ± 0.04 cC	17.64 ± 0.12 bC
	100 mmol/L	38.43 ± 0.01 aB	34.68 ± 0.04 cB	35.29 ± 0.04 bB
	150 mmol/L	52.66 ± 0.13 aA	48.67 ± 0.09 cA	50.68 ± 0.01 bA

^a–c^ Different letters in the same row for each parameter represent statistically significant differences (*p* < 0.05); ^A–C^ different letters in the same column for each parameter represent statistically significant differences (*p* < 0.05).

## Data Availability

Data are contained within the article.
